# The Mental Health Consequences of the Recession: Economic Hardship and Employment of People with Mental Health Problems in 27 European Countries

**DOI:** 10.1371/journal.pone.0069792

**Published:** 2013-07-26

**Authors:** Sara Evans-Lacko, Martin Knapp, Paul McCrone, Graham Thornicroft, Ramin Mojtabai

**Affiliations:** 1 Health Service and Population Research Department, King's College London, Institute of Psychiatry, London, United Kingdom; 2 London School of Economics and Political Science, London, United Kingdom; 3 Department of Mental Health, Johns Hopkins Bloomberg School of Public Health, Baltimore, Maryland, United States of America; UC Davis School of Medicine, United States of America

## Abstract

**Objectives:**

A period of economic recession may be particularly difficult for people with mental health problems as they may be at higher risk of losing their jobs, and more competitive labour markets can also make it more difficult to find a new job. This study assesses unemployment rates among individuals with mental health problems before and during the current economic recession.

**Methods:**

Using individual and aggregate level data collected from 27 EU countries in the Eurobarometer surveys of 2006 and 2010, we examined changes in unemployment rates over this period among individuals with and without mental health problems.

**Results:**

Following the onset of the recession, the gap in unemployment rates between individuals with and without mental health problems significantly widened (odds ratio: 1.12, 95% confidence interval: 1.03, 1.34). This disparity became even greater for males, and individuals with low levels of education. Individuals with mental health problems living in countries with higher levels of stigmatizing attitudes regarding dangerousness of people with mental illness were more vulnerable to unemployment in 2010, but not 2006. Greater agreement that people with mental health problems have themselves to blame, was associated with lower likelihood of unemployment for individuals with and without mental health problems.

**Conclusion:**

These findings study suggest that times of economic hardship may intensify social exclusion of people with mental health problems, especially males and individuals with lower education. Interventions to combat economic exclusion and to promote social participation of individuals with mental health problems are even more important during times of economic crisis, and these efforts should target support to the most vulnerable groups.

## Introduction

Several studies have demonstrated large disparities in unemployment rates between people with and without mental illness. Although most people with mental illness want to work [1], they have higher unemployment rates than people without mental illness and compared to people with other chronic diseases [2]–[4]. High unemployment rates among individuals with mental illness are a major contributor to the substantial societal impact of these disorders [4]–[6]. Unemployment has an impact upon the course and outcome of mental illness [7] and excludes individuals from social participation. A period of macro-economic recession may be particularly difficult for people with mental health problems as they may be at higher risk of losing their jobs and more competitive labour market conditions may make it more difficult for them to find a new job in the first place [8]. This is especially important as research suggests that unemployment could present a specific barrier to recovery from mental illness [9], [10].

Unemployment among people with mental illness may be aggravated during times of economic hardship [7], [11], [12]. Negative attitudes towards marginalized groups (e.g., ethnic minorities or immigrant groups) which often increase during an economic recession [13] are one possible factor influencing this trend. Recent research from Germany suggests that the German public's unwillingness to recommend an individual with depression for a job increased between 2000 and 2011 (i.e., during the period of the economic recession) compared to 1990–2000 [14]. A synthesis of public attitude trends in the US between the 1950s and 1990s showed improvements and declines which mirrored the economic and employment context of the country [7]. Findings regarding the impact of economic recession on disparities [15] and the mechanisms involved, however, are mixed [15]–[18] and we need to better understand the complexity of this relationship. Interestingly, one study [16] did not show that individuals with severe mental illness were at earlier risk of unemployment during times of economic contraction; however, this study specifically investigated individuals with *severe* mental illness who received occupational rehabilitation services and these results may not be broadly generalizable to the wider population of people with mental illness. Furthermore, the effects of the recession since 2008 on disparities are yet to be determined.

In addition to research which suggests that mental health problems increase during times of economic recession, we investigate the impact of the economic recession on people with mental health problems and how this may be mediated by stigma. This paper investigates the impact of economic hardship on unemployment rates of people with mental health problems using Eurobarometer survey data collected from 27 EU countries in 2006 and 2010. We test the hypothesis that the European macro-economic crisis since 2008 has had a greater impact on employment of people with mental health problems compared to people without mental health problems. We also hypothesise that the impact on individuals with mental health problems is greater for people living in regions with greater public stigma towards people with mental illness, after controlling for regional unemployment rates. Additionally, as some research suggests that certain population subgroups, such as men or individuals with low levels of education [19], may be particularly vulnerable during times of economic recession, we investigate whether there is a differential impact of the recession on these subgroups in relation to unemployment.

## Materials and Methods

### Data Source

Full details of the design and sampling for the Eurobarometer surveys *(Eurobarometer Mental Well-being 2006* and *Eurobarometer Mental Health 2010)* are given elsewhere [20], [21]. Data were collected via face-to-face interviews among European Union (EU) citizens (n = 29,248 in 2006 and n = 26,800 in 2010) residing in the 27 member states (approximately 1,000 individuals per country per year). For our analysis we restricted the sample to adults of working age (i.e., 18–64) (n = 20,368 in 2006 and n = 20,124 in 2010).

The initial mental health Eurobarometer survey was conducted in 2006 (fieldwork carried out between 7 December 2005 and 11 January 2006). A second survey assessing attitudes toward mental illness and treatment-seeking was administered to a new sample of respondents in 2010 (between 26 February and 17 March 2010). All participants were recruited via multistage random probability sampling. Participants were representative of residents aged 15 or older in the participating countries.

### Assessments

*Mental health problems* were assessed via the Mental Health Inventory (MHI-5), a well-validated and reliable measure derived from the Short Form 36 (SF-36) [22], [23] As a validated cut-point has not been established for the MHI-5 [22], for the purposes of this study, individuals scoring one standard deviation higher than the standardised mean score were categorised as having mental health problems.

*Stigmatising attitudes* towards individuals with mental health problems were assessed in Eurobarometer 2006 using four questions about various stigmatizing beliefs: (1) People with psychological or emotional health problems constitute a danger to others; (2) People with psychological or emotional health problems are unpredictable; (3) People with psychological or emotional health problems have themselves to blame and (4) People with psychological or emotional health problems never recover. Participants were asked how much they agreed with each statement. Response options were on a 4-point Likert scale from ‘totally disagree’ to ‘totally agree’. Participants who responded ‘totally agree’ or ‘tend to agree’ to each statement were considered as agreeing with that statement. Responses were aggregated within each country to obtain a country-level measure of stigmatizing attitudes.

*Socio-demographic information* included age band (18–29, 30–39, 40–49 and 50–64 years), gender, education level (age at which individuals finished full-time education), and urbanicity (i.e., size of locality of respondent residence: large town, small or middle sized town or rural area/village). Current employment was assessed via the question: ‘What is your current occupation?’ Individuals could endorse the following categories: (1) responsible for ordinary shopping and looking after the home, or without any current occupation, not working (referred to throughout the paper as ‘home-maker’), (2) student, (3) unemployed or temporarily not working, (4) retired or unable to work through illness, or (5) in paid employment.

### National level unemployment rates

National unemployment figures for the years 2006 and 2010 were taken from the Eurostat yearbook (http://epp.eurostat.ec.europa.eu/statistics_explained/index.php/ Europe_in_figures_-_Eurostat_yearbook). Eurostat is a Directorate-General of the European Commission and the statistical office of the European Union. The Eurostat figures for 2006 were moderately highly correlated with the national unemployment rates calculated from the Eurobarometer data (r = 0.76 and 0.70, respectively).

### Statistical Analysis

Four separate multivariable logistic regression models were used to examine predictors of unemployment for individuals with and without mental health problems in 2006 and 2010. Independent variables included age, gender, urbanicity, country-level attitudes regarding dangerousness, recovery, blameworthiness, and unpredictability of people with mental illness. Country-level variables were computed as an average rating for each country and each variable was standardized. Eurobarometer post-stratification weights, based on sex, age, region and size of locality, were used in all analyses to estimate the country-level averages. We used generalized estimating equations (GEE) with the robust variance estimates to model within-country correlations. In the absence of theoretical reasons for specifying a correlation matrix structure, we used an unstructured correlation matrix [24]. In order to investigate whether individual unemployment status differed by population subgroups of interest (i.e., men, individuals with low levels of education and younger individuals) following the recession, we first tested the interaction between survey year and these variables and then tested a three-way interaction between survey year, mental health problems and these variables. All analyses were carried out using SAS version 9.3.

### Ethics statement

Ethical approval was not required as this was secondary data analysis.

## Results

### Socio-demographic characteristics (Table 1)

Compared to individuals without mental health problems, individuals with mental health problems were disproportionately women (χ^2^ = 125.2, df = 1, p<0•001 in 2006 and χ^2^ = 87.9, df = 1, p<0•001 in 2010) and older (χ^2^ = 316.9, df = 3, p<0•001 in 2006 and χ^2^ = 93.9, df = 3, p<0•001 in 2010). The majority of people with and without mental health problems had completed education at least to 16 years of age; however, more of those without mental health problems finished education at age 20+ or were still studying (χ^2^ = 210.1, df = 1, p<0•001 in 2006 and χ^2^ = 237.8, df = 1, p<0•001 in 2010). A higher proportion of people with mental health problems had no formal education or only finished education at 15 years of age (χ^2^ = 313.8, df = 1, p = p<0•001 in 2006 and χ^2^ = 213.7, df = 1, p<•0001 in 2010). Individuals with mental health problems were less likely to be in paid employment or to be a student or home-makers and more likely to be unemployed or disabled/retired, (χ^2^ = 452.6, df = 4, p<0•0001 in 2006 and χ^2^ = 109.4, df = 4, p<0•0001 in 2010).

### Trends in unemployment among people with mental health problems

**Figure 1:**
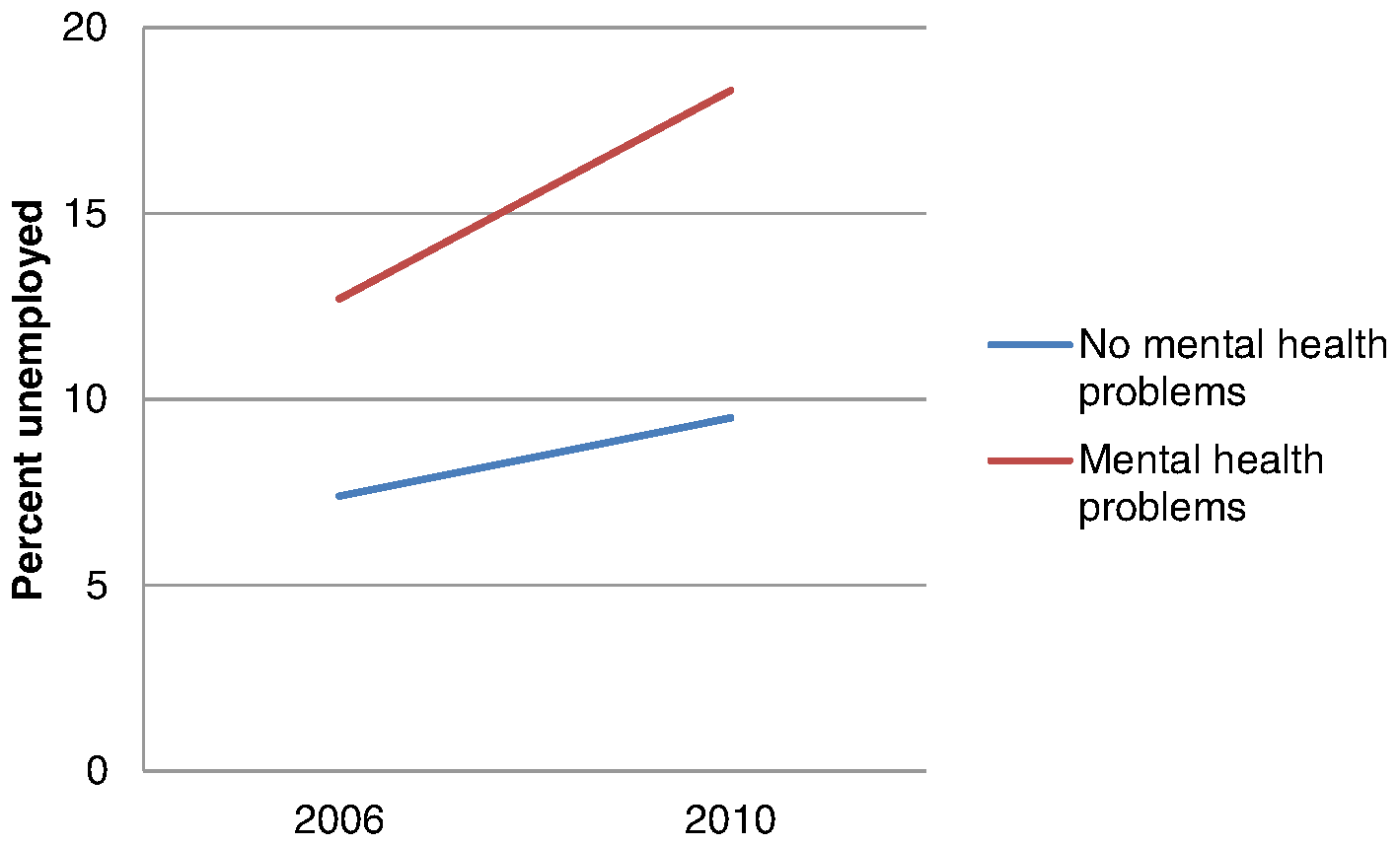
Average unemployment rates among individuals in Eurobarometer 2006 and 2010, stratified by presence of mental health problems (aged 18–65).

Unemployment rates were higher among people with mental health problems compared to those without in both survey years (Table 1). Overall unemployment rates were also higher in 2010 compared to 2006. The gap in unemployment rates between individuals with and without mental health problems widened in 2010 compared to 2006 (Figure 1). The differential trend was statistically significant (odds ratio [OR] for the interaction term for mental health problems by year = 1.12, 95% confidence interval [CI]: 1.03, 1.34. We performed several types of sensitivity analyses to test the robustness of this relationship. We investigated additional cutpoints for individuals scoring in the top ten and the top five percent of mental health problems and their likelihood of unemployment relative to the rest of the population. The differential trend was also statistically significant for these groups: the p-value for the interaction term for mental health problems by year for the top ten percent was 0.020 and the top five percent was 0.018. We also conducted additional sensitivity analyses applying an instrumental variable approach in which individual mental health problems were considered to be endogenous to the model and this also showed a significant relationship and the interaction term for year and mental health problems was also significant (p<0.001).

**Table 1:** Descriptive statistics among people with and without mental health problems in Eurobarometer 2006 and 2010.

	2006				2010			
	Individuals with mental health problems		Individuals without mental health problems		Individuals with mental health problems		Individuals without mental health problems	
	Freq	weighted % 95%CI	Freq	weighted % 95%CI	Freq	weighted % 95%CI	Freq	weighted % 95%CI
Gender								
Male	1183	40.4 (38.6, 42.2)	7728	51.3 (50.5, 52.1)	1668	42.9 (41.3, 44.5)	7759	51.3 (50.5, 52.1)
Female	2166	59.6 (57.8,61.4)	9224	48.7 (47.9, 49.5)	2343	57.1 (55.5, 58.7)	8453	48.7 (47.9, 49.5)
Age group								
18–29	465	16.8 (15.4, 18.3)	4127	28.5 (27.8, 29.3)	679	21.0 (19.6, 22.4)	3716	27.3 (26.6, 28.1)
30–39	622	20.6 (19.1, 22.1)	3828	24.6 (23.9, 25.3)	802	21.8 (20.5, 23.2)	3685	24.0 (23.3, 24.7)
40–49	783	24.7 (23.2, 26.3)	3689	21.9 (21.3, 22.6)	936	24.4 (23.0, 25.8)	3419	21.1 (20.5, 21.8)
50–64	1392	37.8 (36.2, 39.6)	4976	25.0 (24.3, 25.6)	1379	32.8 (31.3, 34.3)	5029	27.6 (26.9, 28.3)
Urbanicity								
Large town	1193	36.5 (34.8, 38.2)	4725	27.5 (26.8, 28.2)	1190	30.8 (29.3, 32.3)	4751	29.5 (28.7, 30.2)
Small or mid-sized town	1177	34.3 (32.6, 35.9)	6170	35.8 (35.1, 36.6)	1356	34.8 (33.3, 36.4)	5680	35.2 (34.4, 35.9)
Rural area	979	29.2 (27.6, 30.8)	6057	36.6 (35.8, 37.4)	1332	34.4 (32.8, 35.9)	5781	35.4 (34.6, 36.1)
Education								
No full time education	83	2.4 (1.9, 3.0)	278	1.61 (1.4, 1.8)	77	1.9 (1.5, 2.4)	171	1.0 (0.9, 1.2)
Education to 15	911	25.2 (24.5, 27.8)	2434	13.1 (12.6, 13.7)	789	19.6 (18.3, 20.8)	1929	11.3 (10.8, 11.8)
Education to 16–19	1574	47.8 (46.0,49.6)	7965	47.1 (46.3, 47.9)	1940	50.3 (48.7, 51.9)	7561	46.0 (45.2, 46.8)
Education to 20+ or still studying	781	24.6 (23.0, 26.1)	6275	38.1 (37.3, 38.9)	1035	28.2 (26.7, 29.7)	6481	41.7 (40.9, 42.5)
Employment								
Long term disabled/Retired	798	21.4 (20.0, 22.8)	1825	8.6 (8.2, 9.0)	663	15.5 (14.3, 16.6)	1869	9.6 (9.2, 10.1)
In paid employment	1584	49.6 (47.9, 51.4)	11042	67.2 (66.5, 67.9)	1862	48.8 (47.2, 50.4)	10200	63.4 (62.6, 64.2)
Student	124	4.5 (3.7, 5.3)	1264	8.6 (8.1, 9.1)	194	6.4 (5.5, 7.3)	1216	9.3 (8.8, 9.8)
Home maker	444	11.9 (10.8, 13.0)	1654	8.5 (8.1, 8.9)	467	11.1 (10.1, 12.1)	1395	7.9 (7.4, 8.3)
Unemployed	399	12.7 (11.4, 13.9)	1167	7.1 (6.7, 7.5)	692	18.2 (17.0, 19.5)	1532	9.8 (9.3, 10.3)

### Relationship between unemployment and mental health status

In each of the survey years, local unemployment rates ascertained by Eurostat were strongly associated with the odds of being unemployed among participants both with and without mental health problems in Eurobarometer (Table 2). Among people with mental health problems, males were more likely to be unemployed than females in 2010 (OR: 1.58, 95% CI: 1.30, 1.92, p<0.001) and marginally more likely to be unemployed than females in 2006 (OR: 1.24, 95%CI: 0.99, 1.57, p = 0.067). The interaction term for gender and year was statistically significant for the entire sample (p<0.001) and among individuals with mental health problems (p<0.01), but not among those without mental health problems. In 2010, 21.7% of men with mental health problems were unemployed, compared to 13.7% in 2006. For women with mental health problems, the difference in unemployment rate between 2010 (15.6%) and 2006 (11.9%) in 2006 was narrower.

**Table 2:** Results of multivariable logistic regression analyses for predictors of unemployment stratified by presence of mental health problems in Eurobarometer 2006 and 2010.

	2006		2010	
Predictors	Individuals with mental health problems Adjusted GEE parameter estimates Odds Ratio (95% CI)	Individuals without mental health problems Adjusted GEE parameter estimates Odds Ratio (95% CI)	Individuals with mental health problems Adjusted GEE parameter estimates Odds Ratio (95% CI)	Individuals without mental health problems Adjusted GEE parameter estimates Odds Ratio (95% CI)
**Individual level variables**				
Gender				
Male	1.24 (0.98, 1.57)	*0.85 (0.74, 0.99)	***1.58 (1.30, 1.92)	1.11 (0.91, 1.36)
Female	1.00 (ref.)	1.00 (ref.)	1.00 (ref.)	1.00 (ref.)
Age group				
18–29	*1.26 (1.05, 1.52)	***1.73 (1.48, 2.01)	*1.25 (1.00, 1.55)	***1.63 (1.42, 1.87)
30–39	1.09, (0.90, 1.32)	1.04 (0.94, 1.16)	*1.22 (1.04, 1.42)	*1.05 (1.00, 1.22)
40–49	1.12 (0.93, 1.34)	*0.86 (0.72, 0.99)	1.01 (0.87, 1.17)	*0.87 (0.77, 0.99)
50–64	1.00 (ref.)	1.00 (ref.)	1.00 (ref.)	1.00 (ref.)
Years of education	***0.73 (0.63, 0.85)	***0.65 (0.59, 0.72)	***0.68 (0.61, 0.75)	***0.55 (0.49, 0.62)
Urbanicity				
Large town	1.03 (0.71, 1.51)	0.93 (0.75, 1.17)	*1.27 (1.00, 1.62)	0.98 (0.84, 1.13)
Small or middle sized town	1.14 (0.85, 1.51)	1.02 (0.88, 1.20)	1.20 (0.95, 1.52)	1.05 (0.88, 1.25)
Rural area	1.00 (ref.)	1.00 (ref.)	1.00 (ref.)	1.00 (ref.)
**Country level variable**				
Dangerousness^a^	1.19 (0.93, 1.54)	1.17 (0.91, 1.51)	*1.41 (1.02, 2.04)	1.12 (0.87, 1.44)
Blame^b^	*0.72 (0.55, 0.93)	*0.78 (0.62, 0.98)	**0.66 (0.50, 0.88)	0.90 (0.71, 1.14)
Unpredictability^c^	1.12 (0.90, 1.40)	1.09 (0.87, 1.37)	0.93 (0.72, 1.20)	0.98 (0.78, 1.24)
Recovery^d^	1.22 (0.91, 1.65)	1.21 (0.97, 1.50)	1.22 (0.90, 1.66)	1.08 (0.81, 1.44)
Unemployment rate 2006	**1.10 (1.02, 1.19)	**1.09 (1.02, 1.16)	1.01 (0.93, 1.10)	0•99 (0.90, 1.08)
Unemployment rate 2010	NA	NA	***1.08 (1.04, 1.12)	***1•09 (1•06, 1.12)

a Average country-level agreeing with the statement: “People with psychological or emotional health problems constitute a danger to others”.

b Average country-level agreeing with the statement: “People with psychological or emotional health problems have themselves to blame.”

c Average country-level agreeing with the statement: “People with psychological or emotional health problems are unpredictable.

d Average country-level agreement with the statement: “People with psychological or emotional health problems never recover.”

* = p<0.05, **p<0.01 ***p<0.005.

In both 2006 and 2010 individuals in the youngest age band (18–29 years), with and without mental health problems, were more likely to be unemployed than individuals in the oldest age band (50–64 years). However, age patterns of unemployment in both survey years varied among those with and without mental health problems in that the younger age was more strongly associated with unemployment among those without mental health problems (p<0.001). Indeed, the unemployed with mental health problems were significantly older than those without mental health problems (mean age = 4.3 vs. 36.1, t-test = 10.16, p = 0.001).

Fewer years of education was significantly associated with unemployment among individuals with and without mental health problems; however, education was more strongly associated with unemployment among individuals with mental health problems compared to those without these problems (p = 0.001). The impact of education on employment was also more substantial during 2010 compared with 2006 among individuals with mental health problems only (p = 0.010). This interaction was also significant among the entire sample (p = 0.020), but not among those without mental health problems.

Urbanicity (i.e., size of the town where participants were recruited) did not play a major role in likelihood of unemployment except that individuals with mental health problems who lived in a large town relative to a rural area were more likely to be unemployed (Table 2), which could be interpreted as implying that a larger labour market disadvantages those with mental health problems.

During 2010, but not 2006, among individuals with mental health problems only, living in a country where a *higher* proportion of the general public agreed that people with mental health problems are dangerous was associated with a higher likelihood of being unemployed (Table 2). During 2006, individuals with mental health problems living in a country where a *lower* proportion of the general public agreed that people with mental health problems have themselves to blame were more likely to be unemployed. This relationship was maintained in 2010. Living in a country where a *higher* proportion of the general public agreed that people with mental health problems will never recover was associated with a marginally higher likelihood of being unemployed among individuals with mental health problems (p = 0.097).

## Discussion

Economic recession has had enormous impacts across much of Europe; however, little information is available about the *specific* impact of the recession on groups who are already vulnerable to social exclusion, specifically individuals with mental illness. This is the first study to demonstrate that the European economic crisis had a greater impact on people with mental health problems, compared to people without mental health problems, as measured by exclusion from employment. Our study also identified important sub-groups which experienced greater impacts of the economic recession in terms of unemployment, specifically men and individuals with low levels of education. Overall, males and individuals with lower levels of education appear to have been affected disproportionately by the recession; both groups had a significantly greater increase in likelihood of being unemployed following the recession. Moreover, for individuals with mental health problems, gender and level of education were particularly important determinants of employment status as the recession seemed to have a disproportionately higher negative impact on their likelihood of being employed for men and those with less education. This may be due to shifts in labour markets: other studies have suggested that men may be more vulnerable to unemployment during the current recession in Europe as they are more likely to work in construction and manufacturing jobs which are more vulnerable to decreases in demand and job loss [25], while other research suggests that this disparity is only evident during the initial stages of a recession [11].

This study also showed that stigmatizing attitudes, specifically beliefs regarding dangerousness of individuals with mental health problems, could be an important mediator in the relationship between unemployment and mental health problems following the recession. Living in a country where a higher proportion of individuals believe that individuals with mental illness are dangerous was associated with a higher likelihood of unemployment for people with mental health problems, but did not influence employment rates for those *without* mental health problems. Moreover, this became significant in 2010, following the economic recession. Other studies have emphasised the persistence of attitudes related to dangerousness and their association with community rejection [26]. Research on racial discrimination suggests that stereotype amplification in relation to risk and fear of victimisation plays an important role in the persistence of racial inequalities and community segregation [27]. These attitudes may be internalised by the stigmatised group. Recent international work underscores the prevalence of experienced and anticipated discrimination among people with depression in relation to employment, suggesting that this is a critical barrier to achieving employment integration [28]. A recent analysis of trends in public attitudes toward people with mental health problems in England and older research from the U.S. also suggested that attitudes to people with mental health problems may harden during periods of economic crisis [7], [29]; however, there is a gap in research around this topic. Surprisingly, a higher proportion of the public endorsing blameworthiness was consistently associated with lower rates of unemployment among people with mental health problems. Previous research has found that stigmatizing attitudes are highly specific in their relation to impact on people with mental health problems. For example, living in a community with stronger beliefs about blameworthiness of individuals with mental illness is associated with lower rates of willingness to seek professional help [30] but also lower levels of perceived discrimination among people with mental health problems [31]. Other research has shown that world views such as stronger just world beliefs for self may be a double edged sword as they are associated with greater blameworthiness; but also lower self stigma among people with mental illness [32]. It could be that environments with greater endorsement of blame and controllability of symptoms and/or illness also engender a context where the guilt and blame associated with those who are not working is increased. Thus, any intervention would need to carefully consider the complexity of cultural factors and beliefs underlying individual and public attitudes.

Previous studies have demonstrated the impact of the recession on public health more generally [33]–[35], however, the selective impact of recession on people with mental health problems, especially males or individuals with lower levels of education, should be acknowledged through both research and policy. Analysis of general government policy responses in Europe following the crisis reveals deficiencies and problems and suggests that governments should allocate resources toward keeping and reintegrating people into employment in addition to initiating programmes that help people cope with the negative effects of job loss to counteract the adverse health effects of the recession [33]. Highlighting the population subgroups who are most vulnerable to economic shocks and identifying ways to mitigate the effects of these shocks is also important. It may be that investment in targeted programmes such as debt advice for people with mental health problems may improve their mental health and financial circumstances [36], [37]. Given the cuts in mental health services across Europe, the impact of the recession is likely to be felt among a growing number of individuals alongside dwindling resources. Lack of resources may strain mental health services during times of higher need leading to decreased access in the face of increased need. In Spain where the impact of the recent recession has been among the greatest, the prevalence of mental disorders diagnosed in primary care settings is increasing. These increases are associated with increases in unemployment and also present among individuals whose employment is threatened and also those who are struggling to make payments on their mortgage [38]. Recent findings from both England and Spain suggest that the recession is associated with a deterioration in population mental health [19], [38]. In addition to people with mental health problems generally, it is important to acknowledge specific subgroups with mental health problems, such as males and those with lower education. In addition to having a higher likelihood of unemployment, these subgroups have lower rates of help-seeking and more negative attitudes about mental illness [29], [39] and thus, may require specific forms of outreach.

### Limitations

This study presents new and important information about the impact of macroeconomic downturn on people with and without mental health problems in Europe using nationally representative data from 27 countries in Europe surveyed over two time points, before and after the onset of the current recession. Nevertheless, the data were not collected with the specific aims of this study in mind and were not longitudinal in nature as the same individuals were not interviewed in the two surveys. Mental health status was determined via a brief self-report measure and thus mental health problems were not verified by a clinician. Additionally, type and severity of problems were not assessed. Most previous research on employment of individuals with mental health problems and also on mental illness stigma has focused on those with severe mental disorders which could not be identified in the Eurobarometer data. Additionally, data on potentially important characteristics such as ethnicity and immigration status or survey response rates were not available. The investigation was limited to two time points only and although the impact of economic recession was clearly evident in 2010, long term effects could not be investigated. Relatedly, as these are observational data, our analyses could not rule out reverse causality, and the potential that people who were unemployed were more likely to develop mental health problems in 2010. Other research has suggested that a large proportion of the consequences of unemployment such as mortality are due to mental health related selection prior to becoming unemployed [40] suggesting that this is an important mechanism to investigate. Our main outcome of interest was unemployment; however, there may be other important effects of the economic crisis in terms of social exclusion which we were not able to examine. As Eurobarometer recruited individuals by household, we were not able to investigate individuals who may have transitioned into more extreme types of exclusion i.e., individuals who became homeless, were in care or hospital settings or were imprisoned. Finally, attitudes about people with mental illness were only collected at one time point in 2006 which precludes assessment of changes in public attitudes over time and its potential impact on unemployment trends. However, the assessment of attitudes preceded the economic crisis and so was not confounded by the effects of the recession.

Past research has consistently shown that people with mental health problems tend to be excluded from employment, housing and social relationships, and that this exclusion has negative social and economic consequences [8]. This study suggests that times of economic hardship are likely to heighten such exclusion for people with mental health problems. The study also provides some preliminary clues as to which groups of individuals with mental health problems are especially vulnerable during times of economic hardship, and what societal factors might moderate this adverse relationship. Use of both individual-level and aggregate-level data to explore this relationship provides new and important evidence about the impact of the macro-social context on individuals during times of economic recession and facilitates micro-macro research in relation to mental health and exclusion [41], [42]. Findings suggest that programmes to combat exclusion and to promote mental health may be more important during times of economic crisis. Future research should examine the long term effects of the economic recession on people with mental health problems and the relationship between different types of employment and social welfare policies and unemployment rates for people with mental health problems.
